# Beyond Extinction: Prolonged Conditioning and Repeated Threat Exposure Abolish Contextual Renewal of Fear-Potentiated Startle Discrimination but Leave Expectancy Ratings Intact

**DOI:** 10.3389/fpsyt.2018.00117

**Published:** 2018-04-06

**Authors:** Arne Leer, Kim Haesen, Bram Vervliet

**Affiliations:** ^1^Clinical Psychology, Utrecht University, Utrecht, Netherlands; ^2^Center for Excellence on Generalization, University of Leuven, Leuven, Belgium

**Keywords:** fear conditioning, extinction learning, habituation, renewal, fear-potentiated startle

## Abstract

Extinction treatments decrease fear via repeated exposures to the conditioned stimulus (CS) and are associated with a return of fear. Alternatively, fear can be reduced via reductions in the perceived intensity of the unconditioned stimulus (US), e.g., through repeated exposures to the US. Promisingly, the few available studies show that repeated US exposures outperform standard extinction. US exposure treatments can decrease fear via two routes: (1) by weakening the CS–US association (extinction-like mechanism), and/or (2) by weakening the subjective US aversiveness (habituation-like mechanism). The current study further investigated the conditions under which US exposure treatment may reduce renewal, by adding a group in which CS–US pairings continued following fear acquisition. During acquisition, participants learned that one of two visual stimuli (CS+/CS−) predicted the occurrence of an aversive electrocutaneous stimulus (US). Next, the background context changed and participants received one of three interventions: repeated CS exposures, (2) repeated US exposures, or (3) continued CS–US pairings. Following repeated CS exposures, test presentations of the CSs in the original conditioning context revealed intact CS+/CS− differentiation in the fear-potentiated startle reflex, while the differentiation was abolished in the other two groups. Differential US expectancy ratings, on the other hand, were intact in all groups. Skin conductance data were inconclusive because standard context renewal following CS exposures did not occur. Unexpectedly, there was no evidence for a habituation-like process having taken place during US exposures or continued CS–US pairings. The results provide further evidence that US exposures outperform the standard extinction treatment and show that effects are similar when US exposures are part of CS–US pairings.

## Introduction

Learning to anticipate future threat can be crucial for survival. When we experience a threat, we extract knowledge about reliable predictors of the threatening event. Future confrontations with these predictors elicit fear and trigger defensive bodily responses. Fear conditioning researchers generally agree that such learning is based on the development of associations between memory representations of these events [e.g., Ref. (1–3)]. These associations are thought to gain strength over successive pairings, such that the predictive stimulus (conditioned stimulus, CS) becomes increasingly successful in activating the memory representation of the aversive event (unconditioned stimulus, US) (3, 4). It follows that the level of CS-elicited fear is a function of (1) the strength of the CS–US association and (2) the intensity of the US represented in memory (5). Formulated in cognitive terms, conditioned fear reflects an interaction between the estimated contingency between CS occurrence and US occurrence and the estimated intensity of this US [see Equation 1 below; (6)]:

Fear=Contingency×Intensity

where Contingency refers to the estimated CS–US contingency, i.e., the probability of US occurrence in the presence of the CS *p*(US|CS) relative to the probability of US occurrence in the absence of the CS *p*(US|noCS) (7), and Intensity refers to the subjective aversiveness of the US rather than its physical intensity.

In order for fear learning to be adaptive, the level of fear should continuously adjust to changes in threat values. According to the formula above, level of fear changes (1) when the estimated CS–US contingency changes and/or (2) when the estimated US intensity changes. Thus, when a CS is no longer followed by the US, the estimated CS–US contingency is expected to lower and fear is expected to decrease. Likewise, when the US is experienced as less aversive than before, the estimated US intensity is expected to lower and fear is expected to decrease.

The first route to fear decrease has received abundant attention. Research extensively showed that repeatedly presenting the CS in the absence of the US does indeed reduce CS-elicited fear (8, 9). This so-called fear extinction procedure has been translated to the treatment of anxiety disorders with considerable success (10–12). Although extinction learning is effective in extinguishing conditioned fear, this type of fear reduction is not permanent. Fear returns when circumstances change, for example following (1) a context change, (2) a time lapse, or (3) sudden unpredicted US presentations [for a review, see Ref. (13)]. These findings suggest that the original CS–US association (the estimated contingency between the CS and the US following aversive learning) persists in memory during extinction learning, forming the basis for the return of fear. Arguably, what is learned during extinction trials is that the lowered CS–US contingency only applies in the current context where extinction learning took place (14). This context dependence of extinction learning limits long-term effectiveness of exposure treatment (15). Therefore, optimizing the robustness of fear reduction is a major impetus for clinical and preclinical research, which has met with mixed levels of success (13).

The second route to fear decrease, via changing the perceived US intensity, has received much less attention, although a number of laboratory studies in healthy individuals confirmed that weakening the perceived US intensity is an effective means to decrease fears (6, 16–18). These studies used different strategies to devalue the US such as (1) repeated exposures to the actual US, (2) exposures to USs of decreasing intensity, and (3) instructions to recall the aversive event and mentally rescript it into a more neutral image (imagery rescripting). Interestingly, some studies also suggest that fear decrease obtained by changing the perceived US intensity may be more resistant to context changes than the standard fear extinction effect (6, 16, 18). Thus, when CS–US fear conditioning occurred in context A and was followed by repeated US presentations in a separate context B, test presentations of the CS in context A elicited a low level of fear as measured by skin conductance reactivity (6). Interestingly, self-reported estimations of US occurrence upon CS exposures remained high at test, suggesting that the observed fear reduction during test presentations of the CS resulted from a reduction in estimated US intensity rather than the estimated CS–US contingency. This would imply that targeting US intensity estimations via US devaluation procedures has more potential for achieving context-independent fear reduction in the treatment of anxiety.

The current study had two goals. First, we aimed to replicate the contextual renewal of fear extinction and the context-independent fear reduction of US exposures using a physiological measure that is sometimes regarded as a more accurate index of fear [startle reflex modulation, see Ref. (19, 20)]. Second, we aimed to further specify the mechanism behind the context-independent fear reduction effect of US exposures. US exposures can decrease fear via the two fear reduction routes described earlier: lowering the perceived US intensity or lowering the perceived CS–US contingency. In the first case, mere repeated exposure to the US engages processes that oppose the US’s negative emotional impact and thus its intensity (21). As a result, the CS now predicts the occurrence of a less aversive US and fear decreases. In the second case, the experience of the US in the absence of the CS [*p*(US|noCS)] changes the estimated correlation between both events (CS–US contingency). When the US occurs equally or more often in the absence of the CS than in its presence, the CS no longer reliably predicts the occurrence of the US and CS-elicited fear decreases (7, 22).

One way to disentangle two mechanisms is by blocking one while examining the other. For that purpose, the current study investigated the fear reduction effect and context-dependency of continued CS–US pairings. This should strengthen rather than weaken the CS–US contingency (controlling for reductions in the estimated CS–US contingency), while still providing opportunity for US habituation via the continued US presentations. It was shown a long time ago that continued CS–US pairings can effectively weaken conditioned reactions [“inhibition with reinforcement” (23), see Ref. (24), for a review]. Hovland (25) confirmed these effects in a human fear conditioning procedure, reporting that CS–US pairings initially led to increased but eventually decreased conditioned skin conductance reactivity in human subjects. To date, countering this type of response habituation is a major challenge in human fear conditioning studies; pilot work is often needed to fine-tune parameters that ensure persistent conditioned responding throughout a CS–US conditioning procedure (e.g., by adjusting the CS–US reinforcement schedule). Despite the generality of this phenomenon, continued CS–US pairings have received no attention as a fear reduction method in humans. Here, we investigated its potential to produce context-independent fear reduction in humans. This would specify the mechanism underlying the previously reported US exposures effects and could lead to the counter-intuitive conclusion that continued fear conditioning is a more effective means for reducing fears in humans than standard fear extinction.

## Materials and Methods

### Participants

Based on a power analysis [*f*  = 0.23, cf. Haesen and Vervliet (6), required *n* = 51], and anticipating potentially noisy psychophysiological data, the sample size was set at *n* = 60. Sixty-six psychology students and community volunteers participated in return for payment (8 euros) or course credits. Participants were randomly assigned to one of only three groups and gave written informed consent in accordance with the Declaration of Helsinki, after they were informed that they could abort the experiment at any time. *A posteriori* six participants were excluded because of flawed probe presentations (*n* = 3) or because shocks were presented at an unnoticed intensity (*n* = 3). This resulted in a sample of 60 participants (49 women, mean age = 22.43 years, *SD* = 4.03): group CS-only, *n* = 21; group US-only, *n* = 20; group CS–US, *n* = 19. The Social and Societal Ethics Committee of KU Leuven approved the study.

### Apparatus

Stimulus presentation, stimulus sequence, intertrial intervals (ITIs), and response registration were controlled by Affect 4.0 software (26).

#### Conditioned Stimuli and Contexts

Two conditioned stimuli [a square and a triangle, CS+, i.e., occasionally followed by shock (see Procedure), and CS−, i.e., never followed by shock, or *vice versa*, depending on the counterbalancing scheme] were presented on a computer screen (Dell LCD monitor, type 1707 FPc). The gray shapes with black borders were presented in a white frame. To manipulate the context, the background color of the computer screen alternated between orange (RGB: 255,153,51) and blue (RGB: 136,196,255).

#### Unconditioned Stimulus

A 2-ms electrocutaneous stimulus served as US. The stimulus was administered to the wrist of the dominant hand by a Digitimer DS7A constant current stimulator (Hertfordshire UK) via a pair of V91-01-8 mm reusable Bilaney Ag/AgCL electrodes, filled with K-Y Jelly (Johnson & Johnson).

#### Skin Conductance Reactivity

To record skin conductance responses (SCRs), a pair of 8-mm Ag/AgCl electrodes were attached to the hypothenar palm of the dominant hand. A skin conductance coupler (manufactured by Coulbourn Instruments, model V71-23) applied a constant voltage of 0.5 V across these electrodes. The skin conductance signal passed through a Labmaster DMA 12 bit analog-to-digital converter (Scientific Solutions, Solon, OH, USA) and was digitized at 10 Hz from 2 s prior to CS onset until 6 s after CS offset.

#### Fear-Potentiated Startle (FPS)

We registered oculi electromyographic activity (EMG) through three 0.25-cm diameter Ag/AgCl electrodes filled with TECA electrolyte. The skin under the eyes and on the forehead was cleaned with micellar water (Avène, Pierre Fabre) to reduce inter-electrode resistance. Next, the electrodes were placed on the left side of the face according to site specifications proposed by Fridlund and Cacioppo (27). The raw signal was amplified by a Coulbourn isolated bioamplier with a bandpass filter (V75-04). The recording bandwidth of the EMG signal was between 10 and 20 Hz (3 dB). The signal was rectified online and smoothed by a Coulbourn multifunction integrater (V76-23A) with a time constant of 50 ms. The startle probe was a 102 dB(A) 50-ms burst of white noise, presented through stereo headphones. The signal was measured 100 ms before probe onset at 1,000 Hz.

#### US Expectancy

We measured trial-by-trial subjective shock expectancy with an 11-point visual analog scale (VAS) that appeared at the bottom of the screen on every trial. The VAS ranged from 0 to 10 and was labeled: 0 = *certainly no shock*, 5 = *maybe*, and 10 = *certainly a shock*. Participants registered their rating using a left mouse click.

#### US Aversiveness

At the start and end of the experiment, participants were asked to verbally rate the aversiveness of the shock with a number ranging from 0 (=*not aversive*) to 10 (=*very aversive*).

### Procedure

Participants were welcomed into the lab and gave informed consent. After the electrodes were fitted, the shock intensity was set to a level the participant experienced as “definitely uncomfortable but not painful” via a standard shock workup procedure [cf. Ref. (28)]. The participant was asked to rate the aversiveness of the selected shock intensity. Next, the experimenter instructed participants about their task. They were informed that geometrical shapes that could be followed by shock would appear on the computer screen and that it was participant’s task to predict the occurrence of the shock.

The experimenter further instructed participants how to use the US expectancy VAS. The experiment consisted of five phases (see Table 1; note that phases 3–5 are illustrated). The first two phases served to optimize the conditions for the psychophysiological measurements. Phase 1, the probe habituation phase, consisted of eight presentations of the startle probe at random moments (10.5–11.5 s between two probe presentations) to decrease initial startle responses to the startle probe. Phase 2, the pre-acquisition phase, consisted of a non-reinforced presentation of the two CSs, in random order, to reduce initial orienting responses to these stimuli. Next, during the acquisition phase (phase 3) the two CSs were each presented four times, in semi-random order (i.e., no more than two similar stimulus presentations in succession), against an orange or blue background (depending on the counterbalancing scheme); CS+ was always followed by shock, CS− never. Following acquisition, the background changed color. In the following intervention phase, the crucial manipulation took place. Participants were randomly assigned to one of only three groups. Participants in the first group, group “CS-only,” received eight non-reinforced CS-only presentations of each stimulus. Participants in the second group, group “US-only,” received eight US-only presentations. The remaining participants in the “CS-US-paired” group received additional acquisition trials: eight reinforced CS+ presentations and eight non-reinforced CS− presentations, in semi-random order. During the subsequent test phase, the background color changed back to the color used during acquisition and both CSs were each presented four times without shock and in semi-random order. The order of context and the geometrical shapes serving as CS+ and CS− were counterbalanced. At the end of the experiments, participants were again asked to rate the aversiveness of the shock.

**Table 1:** Overview of the experimental phases and groups.

	Acquisition	Intervention	Test
CS-only	4 CS+/US	8 CS+	4 CS+
	4 CS−	8 CS−	4 CS−
US-only	4 CS+/US	8 US	4 CS+
	4 CS−		4 CS−
CS-US-paired	4 CS+/US	8 CS+/US	4 CS+
	4 CS−	8 CS−	4 CS−

*CS+ and CS− are geometrical shapes (counterbalanced); US is a mild aversive electrical shock. The background coloring refers to the experimental context (blue or orange background of the computer screen, counterbalanced)*.

Trials comprised the presentation of a CS and lasted for 8 s. A startle probe was presented on each trial 5.5–6.5 s (*M* = 6 s) following CS onset. Shock was delivered 7.5 s following CS+ onset. At the onset of each trial, the VAS appeared at the bottom of the screen. The VAS disappeared after participants confirmed their rating with a mouse click or at CS offset. In between trials, there were ITIs that lasted for 24.5–25.5 s (*M* = 25 s). A “noise alone” (NA) startle probe was delivered on half of the ITIs, 12 s following CS offset and at least 12 s before the next CS onset. These NA probes were included following the recommendations by Lissek et al. (29) to illustrate baseline levels of responding. In the US exposure group, and only during the intervention phase that was devoid of CS presentations, shocks were delivered 32.5–33.5 s apart (*M* = 33 s) and probes were delivered 1–2 s (*M* = 1.5 s) before each shock presentation. In order to level the number of probe presentations across the three experimental groups, 16 inter-trial startle probes were added to the intervention phase of the US exposure group only.

### Data Reduction and Statistical Approach

Skin conductance response data of two participants and EMG data of four participants were substantially noisy and therefore discarded from the analyses. This resulted in 58 participants providing SCR data (group CS-only: *n* = 19; group US-only: *n* = 20; group CS–US: *n* = 19) and 56 participants providing startle EMG data (group CS-only: *n* = 18; group US-only: *n* = 19; group CS–US: *n* = 19).

Skin conductance responses and startle EMG responses were extracted with Psychophysiological Analysis software (30). SCR amplitudes were calculated by determining the peak of the skin conductance level in the time interval between CS onset and 6,000 ms after CS onset (31, 32). A baseline SCR (calculated as the average skin conductance level 2,000 ms prior to CS onset) was subtracted from the calculated peak. Amplitudes smaller than 0.02 μS were scored as 0 (33). Next, a range correction [SCR/SCR_MAX_; (34)] and square-root transformation were applied (33). FPS amplitudes were calculated by determining the maximum EMG value within 21–175 ms after stimulus onset. This maximum was subtracted by the EMG baseline calculated by averaging EMG scores 0–20 ms after stimulus onset. To minimize interindividual differences, SCR and FPS amplitudes were *T*-transformed [*T* = 50 + 10 × [(raw score − *M*)/*SD*]; (35); cf (6).].

ANOVAs that tested between-subject effects were performed on data that were *T*-transformed over all trials except the intervention trials. This was done because (1) group US-only did not provide CR data during the intervention phase while the other two groups did—leaving out the intervention trials resulted in an equal amount of trials across the three groups, and (2) group differences in responding were expected during the intervention phase, and hence using the data from this phase to calculate *T*-scores may cause between-group differences in the test phase unrelated to group differences that were hypothesized to arise from the experimental manipulations. For ANOVAs that tested within-subject effects, i.e., examining the intervention phase data, values were *T*-transformed over all trials. Follow-up *t*-tests were always performed on the same data set that was used for the preceding ANOVA.

To test whether the US-only and the CS-US-paired interventions affected the US value, SCR amplitudes in response to the US were calculated by subtracting the baseline (average SCR from 2,000 ms before CS onset) from the peak SCR in the time interval between 1 s following and 6.5 s following shock. The resulting amplitudes were *T*-transformed over all intervention trials.

Context renewal is typically assessed by testing the increase in fear to a conditioned-and-extinguished stimulus upon chances in the background context (36). Because our US-only and CS–US paired groups were not subjected to a traditional extinction protocol, context renewal could only be directly tested in the CS-only group. The hypothesis that US-only exposures and prolonged CS–US exposures, as opposed to CS-only exposures, reduce fear renewal was therefore tested by comparing the three groups on differential responding during the test phase.

## Results

Following the recommendations by Field (37), when the sphericity assumption was violated, Greenhouse–Geisser (0.70 ≤ ε ≤ 0.75) or Huynh–Feldt (ε > 0.75) corrections were applied; in case of severe violations (ε < 0.70), Pillai’s Trace test statistic is reported. To control for Type I error rates for multiple comparisons, Bonferroni corrections were applied to all 13 *post hoc t*-tests (α = 0.05/13 = 0.004). All other effects were tested at α = 0.05.

### US Expectancy

#### Acquisition Phase

**Figure 1:**
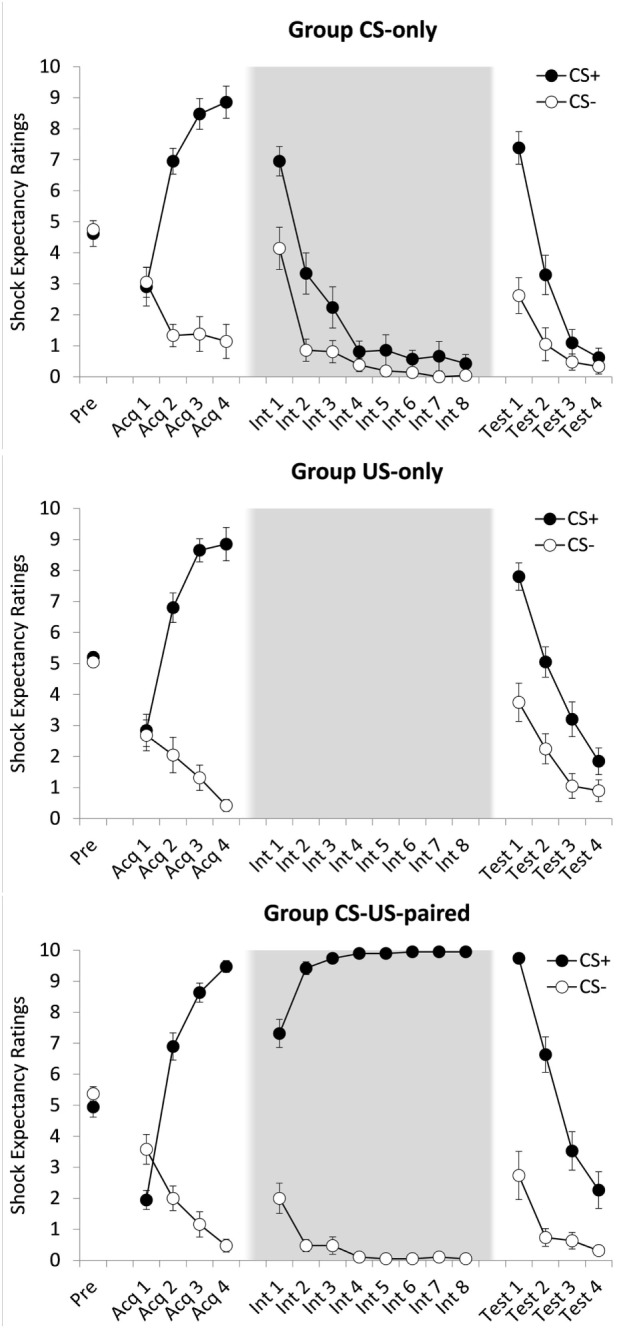
Mean shock expectancy ratings for groups CS-only, US-only, and CS-US-paired as a function of stimulus type (CS+/CS−) and trial. Acq, acquisition trial; Int, intervention trial; Test, test trial. All groups received one CS-pre-exposure trial and four acquisition trials. Four test trials followed eight CS-alone presentations (group CS-only) eight US-alone presentations (group US-only) or eight continued CS–US pairings (group CS–US paired). Background colors represent the experimental contexts. Error bars represent SEMs.

A 2(CS: CS+ vs. CS−) × 4(Trial: 1–4) × 3(Group: CS-only vs. US only vs. CS-US-paired) ANOVA showed a main effect of CS, *F*(1, 54) = 266.95, *p* < 0.001, $\eta_{p}^{2}=0.\text{832}$, and of Trial, Pillai’s Trace = 0.832, *F*(3, 52) = 32.16, *p* < 0.001, $\eta_{p}^{2}=0.\text{65}0$. These effects were qualified by a CS × Trial interaction, Pillai’s Trace = 0.837, *F*(3, 52) = 88.71, *p* < 0.001, $\eta_{p}^{2}=0.\text{837}$, with US expectancy ratings increasing over time for CS+, but not for CS− (see Figure 1). There were no other effects, *F* ′s < 1. *Post hoc* paired samples *t*-tests revealed no differences in US expectancy between the first CS+ trial and the first CS− trial, *t*(57) = 1.46, *p* = 0.149 (α = 0.004), and higher US expectancy during the fourth CS+ trial compared to the fourth CS− trial, *t*(58) = 20.34, *p* < 0.001 (α = 0.004), *d_z_* = 2.648. In sum, acquisition of differential US expectancy was successful and comparable across the three groups.

#### Intervention Phase

To test whether CS exposures resulted in extinction of US expectancy, a 2(CS) × 8(Trial: 1–8) ANOVA was conducted on the data provided by group CS-only. There were main effects of CS, *F*(1, 20) = 7.96, *p* = 0.011, $\eta_{p}^{2}=0.\text{285}$, and Trial, Pillai’s Trace = 0.945, *F*(7, 14) = 34.69, *p* < 0.001, $\eta_{p}^{2}=0.\text{945}$, which were qualified by a CS × Trial interaction, Pillai’s Trace = 0.589, *F*(7, 14) = 2.86, *p* = 0.045, $\eta_{p}^{2}=0.\text{589}$. Over the course of this phase US expectancy ratings dropped at a steeper rate for CS+ than for CS−. A *post hoc* paired samples *t*-test revealed no differences in mean ratings between the two CS types during the last extinction trial, *t*(20) = 1.32, *p* = 0.202 (α = 0.004), suggesting successful extinction of differential US expectancy. A similar analysis on the data of group CS-US-paired was run to examine the effects of prolonged conditioning. There was a main effect of CS, *F*(1, 18) = 2,239.39, *p* < 0.001, $\eta_{p}^{2}=0.\text{992}$, but not of Trial, *F*(7, 126) = 1.14, *p* = 0.345, and a CS × Trial interaction, *F*(7, 126) = 32.45, *p* < 0.001, $\eta_{p}^{2}=0.\text{643}$, reflecting an increase in differential responding over the course of this phase (see Figure 1). As expected, at the end of this phase, ratings remained higher for CS+ than for CS−, *t*(18) = 94.00, *p* < 0.001 (α = 0.004), *d_z_* = 21.565.

#### ABA Renewal

First, we tested whether the widely reported ABA renewal effect was replicated. To this end a 2(CS) × 2(Trial: last intervention phase trial vs. first test phase trial) ANOVA on group CS-only was conducted. There were main effects of CS, *F*(1, 20) = 37.71, *p* < 0.001, $\eta_{p}^{2}=0.\text{653}$, and Trial, *F*(1, 20) = 348.74, *p* < 0.001, $\eta_{p}^{2}=0.\text{946}$, which were qualified by a CS × Trial interaction, *F*(1, 20) = 14.85, *p* < 0.001, $\eta_{p}^{2}=0.\text{426}$. Increases over time were steeper for CS+ than for CS−. During the first test trial, US expectancy was higher to CS+ than to CS−, *t*(20) = 4.98, *p* < 0.001 (α = 0.004), *d_z_* = 1.087, indicating that our standard extinction procedure was associated with ABA renewal.

#### Test Phase

Next, we compared the three groups on strength of differential responding during the test phase. A 2(CS) × 4(Trial: 1–4) × 3(Group) ANOVA yielded main effects of CS, Pillai’s Trace = 0.651, *F*(1, 57) = 106.40, *p* < 0.001, $\eta_{p}^{2}=0.\text{651}$, Trial, Pillai’s Trace = 0.888, *F*(3, 55) = 145.52, *p* < 0.001, $\eta_{p}^{2}=0.\text{889}$, and Group, *F*(2, 57) = 8.27, *p* = 0.001 $\eta_{p}^{2}=0.\text{225}$, and interaction effects of CS × Trial, Pillai’s Trace = 0.584, *F*(3, 55) = 25.72, *p* < 0.001, $\eta_{p}^{2}=0.\text{584}$, and CS × Group, *F*(2, 57) = 6.67, *p* = 0.003, $\eta_{p}^{2}=0.\text{19}0$, but not of Trial × Group, Pillai’s Trace = 0.132, *F*(6, 112) = 1.32, *p* = 0.254. All these effects subsumed under a significant three-way interaction, Pillai’s Trace = 0.278, *F*(6, 112) = 3.01, *p* = 0.009, $\eta_{p}^{2}=0.\text{139}$. Follow-up analyses revealed that the strength of differential responding was comparable across the three groups during the first test trial, CS × Group, *F*(2, 57) = 2.96, *p* = 0.060, $\eta_{p}^{2}=0.0\text{94}$, but not during the last test trial, *F*(2, 57) = 4.39, *p* = 0.017, $\eta_{p}^{2}=0.\text{133}$. There was evidence for sustained differential responding at the end of the test phase for groups CS-US-paired (CS+, *M* = 2.26, *SD* = 2.58; CS−, *M* = 0.32, *SD* = 0.67), *t*(18) = 3.42, *p* = 0.003 (α = 0.004), *d_z_* = 0.785, but not for group US-only (CS+, *M* = 1.85, *SD* = 1.93; CS−, *M* = 0.90, *SD* = 1.55), *t*(19) = 2.41, *p* = 0.026 (α = 0.004), or for group CS-only (CS+, *M* = 0.62, *SD* = 1.40; CS−, *M* = 0.33, *SD* = 1.11), *t*(20) = 1.83, *p* = 0.083 (α = 0.004), reflecting stronger inhibitory trace formation in the latter groups.

### Skin Conductance Responses

#### Acquisition Phase

A 2(CS) × 2(Block: 1 vs. 2) × 3(Group) ANOVA yielded a main effect of CS, *F*(1, 55) = 42.32, *p* < 0.001, $\eta_{p}^{2}=0.\text{435}$, indicative of successful acquisition of differential SCRs. There were no other effects, CS × Block, *F*(1, 55) = 2.06, *p* = 0.157, all other *F* ′s < 1.46, largest *p* = 0.242.

#### Intervention Phase

**Figure 2:**
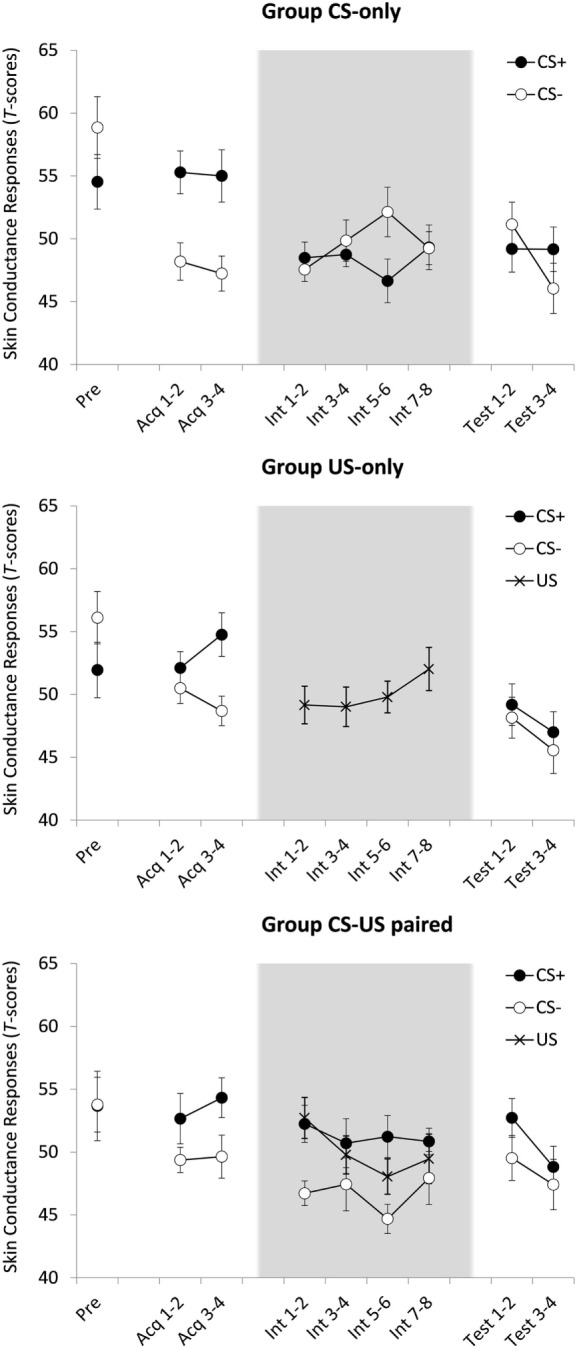
Mean standardized skin conductance responses (SCRs) for groups CS-only, US-only, and CS-US-paired as a function of stimulus type (CS+/CS−/US) and trial. Acq, acquisition trial; Int, intervention trial; Test, test trial. All groups received one CS-pre-exposure trial and four acquisition trials. Four test trials followed eight CS-alone presentations (group CS-only) eight US-alone presentations (group US-only) or eight continued CS–US pairings (group CS-US-paired). Background colors represent the experimental contexts. Error bars represent SEMs. Note that the data in this plot represent SCR magnitudes standardized across all test trials. These data were only used to test within-subject effects. When between-subject effects were tested acquisition and test data were T-transformed over all trials but the intervention trials (since the content of these trials was manipulated between groups). For groups US-only and CS-US-paired, the calculated magnitudes in response to US presentations during the intervention were T-transformed over all US amplitudes during intervention.

Figure 2 suggests that in group CS-US-paired, acquired differential responding generalized to the new context, i.e., to the first block of the intervention phase. This was indeed the case, *F*(1, 18) = 8.90, *p* = 0.008, $\eta_{p}^{2}=\text{331}$. Unexpectedly, however, in group CS-only, there was no evidence for such generalization of acquisition, *F* ′s < 1. The results of 2(CS) × 4(Block: 1 vs. 2 vs. 3 vs. 4) ANOVAs will be reported for both groups nevertheless. Group CS-only showed no main effect of CS, *F*(1, 18) = 1.75, *p* = 0.203, nor was there a main effect of Block, *F* < 1, nor a CS × Block interaction, *F*(3, 54) = 2.17, *p* = 0.110. Group CS-US-paired showed a main effect of CS, *F*(1, 18) = 6.21, *p* = 0.023, $\eta_{p}^{2}=0.\text{256}$, meaning that participants continued giving stronger responses to CS+ than to CS− during this phase. The effects of Block and CS × Block were not significant, *F* ′s < 1. Thus, the SCR data did not provide evidence for inhibition with reinforcement.

#### ABA Renewal

A 2(Phase: intervention vs. test) × 2(CS) × 2(Block) ANOVA comparing the last two intervention phase blocks with the two test phase blocks was conducted on the data of group CS-only. No effects reached statistical significance, *F* ′s < 1, meaning that there was no evidence for ABA renewal of electrodermal responding.

#### US Habituation

A 4(Block) × 2 (Group; US-only and CS-US-Paired) ANOVA revealed no main effect of Block and no Block × Group interaction effect, all *F* ′s < 1.04, all *p* ′s < 0.39. This means that unconditioned responding did not decrease over consecutive US-only or paired CS–US trials.

#### Test Phase

Given that ABA renewal was absent in the SCR data of the CS-only group, the test phase data of the other two groups should be interpreted with caution. An ANOVA comparing the three groups on differential responding during the two test phase blocks showed a main effect of Block, *F*(1, 55) = 7.74, *p* = 0.007, $\eta_{p}^{2}=0.\text{123}$, with lower SCRs in test block 2 compared to test block 1. There was no main effect of CS, *F*(1, 55) = 2.87, *p* = 0.096, all other *F* ′s < 1.219, largest *p* = 0.303. Thus, regardless of group, there was no evidence for differential responding in this phase.

### Startle EMG Responses

#### Acquisition Phase

**Figure 3:**
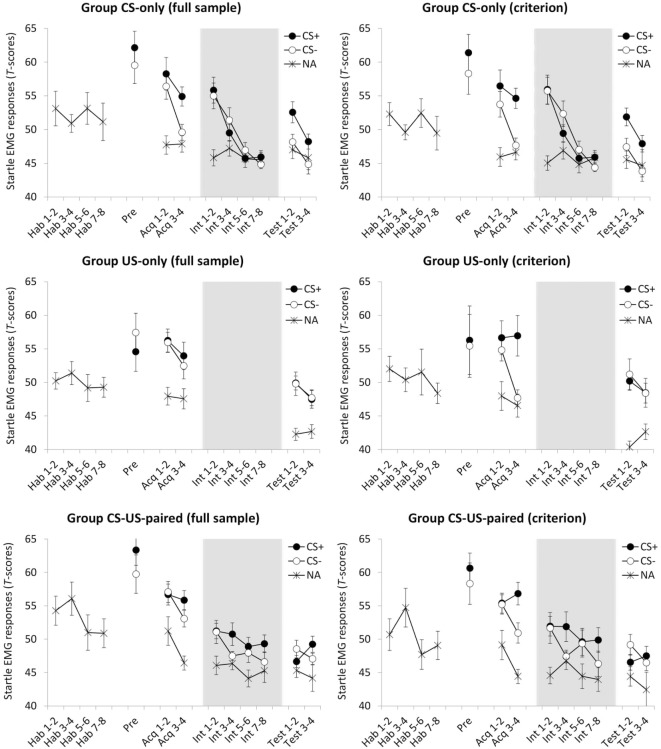
Mean standardized fear-potentiated startle (FPS) responses for groups CS-only, US-only, and CS-US-paired as a function of stimulus type (CS+/CS−/NA) and trial, presented for the full sample (left panel) and for the criterion sample (right panel; only including participants that showed differential startle EMG responding during the last acquisition block). Hab, habituation trial; Pre, pre-test trial Acq, acquisition trial; Int, intervention trial; Test, test trial; NA, noise alone, i.e., startle probe presentation during half of the intertrial intervals. All groups received eight probe habituation trials, one CS-pre-exposure trial and four acquisition trials. Four test trials followed eight CS-alone presentations (group CS-only) eight US-alone presentations (group US-only) or eight continued CS–US pairings (group CS-US-paired). Background colors represent the experimental contexts. Error bars represent SEMs. Note that the data in this plot represent FPS magnitudes standardized across all test trials. These data were only used in ANOVAs that tested within-subject effects. ANOVAs that tested between-subject effects were performed on data that were T-transformed over all trials except the intervention trials (since the content of these trials was manipulated between groups). Further note that, because the timing of startle probes during the US-only intervention was different from the other groups, and therefore not comparable, they are not presented.

A 2(CS) × 2(Block) × 3(Group) ANOVA showed main effects of CS, *F*(1, 53) = 4.18, *p* = 0.046, $\eta_{p}^{2}=0.0\text{73}$, and Block, *F*(1, 53) = 15.54, *p* < 0.001, $\eta_{p}^{2}=0.\text{227}$. Responses were stronger during CS+ and habituated over time. Figure 3 suggests that differential responding increased over time, but the CS × Block interaction did not reach statistical significance, *F*(1, 53) = 3.62, *p* = 0.063. No other effects were found, *F* ′s < 1, suggesting that acquisition of differential startle EMG responding was of comparable strength across the three groups. However, whereas Figure 3 convincingly shows differential responding in group CS-only, it does not for the other two groups, and the absence of a CS × Group interaction may well be due to a power issue. Furthermore, planned comparisons (α = 0.05) testing for differential responding during the final acquisition block revealed a significant effect in group CS-only, *t*(17) = 3.51, *p* = 0.003, *d_z_* = 0.827, but not in group US-only, *t* < 1, and group CS-US-paired, *t*(18) = 2.043, *p* = 0.056. Because testing the hypothesis requires that all groups show successful and comparable fear learning, analyses were rerun only with participants that showed any differentiation in startle EMG responses during the last acquisition block (criterion: CS+ >CS−. In this criterion sample (see the right panel of Figure 3; group CS-only, *n* = 16/18; group US-only, *n* = 11/19; group CS-US-paired, *n* = 13/19), the ANOVA demonstrated main effects of CS, *F*(1, 37) = 22.98, *p* < 0.001, $\eta_{p}^{2}=0.\text{382}$, and Block, *F*(1, 37) = 9.05, *p* = 0.005, $\eta_{p}^{2}=0.\text{197}$. There was also a CS × Block interaction, *F*(1, 37) = 14.43, *p* < 0.001, $\eta_{p}^{2}=0.\text{281}$, meaning that differential responding increased over time. No other effects were found, *F* ′s < 1. Planned comparisons (α = 0.05) further revealed differential responding during the final acquisition block in all groups: CS-only, *t*(15) = 5.97, *p* < 0.001, *d_z_* = 1.493; US-only, *t*(10) = 2.99, *p* = 0.014, *d_z_* = 0.902; CS-US-paired, *t*(12) = 5.62, *p* < 0.001, *d_z_* = 1.559.

Still, one may argue that also in the criterion sample the absence of CS × Group and CS × Block × Group interactions may have resulted from low statistical power. Ideally, one would want to assess whether *H*_0_ (i.e., there are no between-group differences) is better supported by the observed data as compared to *H*_A_ (i.e., there are between-group differences). This cannot be achieved with traditional null hypothesis testing. Therefore, we applied Bayesian hypothesis testing, which serves this very purpose (38). In Bayesian hypothesis testing, Bayes factors (BFs) are used to quantify the relative support of competing hypotheses, e.g., a BF of 1 means that there is equal support for the different hypotheses under investigation (39). Note that we used the JASP statistical software package (40) that produces a slightly different BF after each rerun and therefore we report “BF ≈” instead of “BF =” [cf. Ref. (38)].

First, to test the probability of the data assuming the CS × Group interaction, we compared a non-interaction model with main effects of CS and Group (M_NI_, where NI refers to no interaction) to a full model containing both main effects and the CS × Group interaction effect (M_F_, where F refers to full). The results of the Bayesian analyses show that it was 5.53 times more probable, BF_MNI/MF_ ≈ 5.53, that there was no CS × Group interaction than that there was one. Next, to test the probability of the data assuming a CS × Block × Group interaction, we compared a model with main effects of CS, Block, and Group, including all two-way interactions, but excluding the three-way interaction (M_NTI_, where NTI refers to no three-way interaction) to a full model also including the three-way interaction (M_F_, where F refers to full). Likewise, this Bayesian analysis showed that it was 5.09 times more probable, BF_MNTI/MF_ ≈ 5.09, that there was no CS × Block × Group interaction than that there was one.

In sum, the Bayesian statistical approach provided support that in our criterion sample acquisition of differential startle EMG responding was comparable across the three groups. The below reported analyses were therefore conducted on the criterion sample. It is of note that the below analyses run on the data of the full sample returned similar results.

#### Intervention Phase

To test the effects of CS exposure and prolonged CS–US exposure, respectively, responding over the course of the intervention phase was examined. First, although unexpected, Figure 3 suggests that in both groups differential responding was gone immediately following the first context switch. This was confirmed by *post hoc* comparisons, *t* ′s < 1. Hence, for group CS-only, possible elimination of differential responding at the end of this phase could not be attributed to extinction. Next, for each group, we ran a separate 2(CS) × 4(Block) ANOVA. In group CS-only, there was a main effect of Block, Pillai’s Trace = 0.793, *F*(3, 13) = 16.69, *p* < 0.001, $\eta_{p}^{2}=0.\text{793}$, reflecting habituation over time. There were no effects of CS, *F* < 1, or CS × Block, *F*(3, 45) = 1.36, *p* = 0.267. In group CS-US-paired, there were no effects of CS, *F*(1, 12) = 2.39, *p* = 0.148, and Block, *F*(3, 36) = 1.49, *p* = 0.233, and there was no interaction effect, *F* < 1. Thus, there was no evidence for continued differential responding (pointing toward inhibition with reinforcement) or for habituation over time as a result of prolonged CS–US exposure.

#### ABA Renewal

A 2(Phase: intervention vs. test) × 2(CS) × 2(Block) ANOVA comparing the last two intervention phase blocks with the two test phase blocks was conducted on the data of group CS-only. There were main effects of Phase, *F*(1, 15) = 20.50, *p* < 0.001, $\eta_{p}^{2}=0.\text{577}$, CS, *F*(1, 15) = 8.98, *p* = 0.009, $\eta_{p}^{2}=0.\text{375}$, and Block, *F*(1, 15) = 10.99, *p* = 0.005, $\eta_{p}^{2}=0.\text{423}$. There were no interaction effects of Phase × Block, *F*(1, 15) = 2.05, *p* = 0.173, CS × Block, *F* < 1, or Phase × CS × Block, *F*(1, 15) = 2.42, *p* = 0.141. The crucial Phase × CS interaction, however, was significant, *F*(1, 15) = 8.27, *p* = 0.012, $\eta_{p}^{2}=0.\text{355}$. Follow-up *t*-tests, using the average of the four trials, showed no evidence for differential responding during the last half of the intervention phase, *t* < 1, and revealed renewal of differential responding during the test phase, *t*(15) = 3.89, *p* = 0.001 (α = 0.004), *d_z_* = 0.972.

#### Test Phase

To compare the groups on responding during the test phase, a 2(CS) × 2(Block) × 3(Group) ANOVA was conducted. There was a main effect of Block, *F*(1, 37) = 7.70, *p* = 0.009, $\eta_{p}^{2}=0.\text{172}$, indicating habituation over time, but not of CS, *F*(1, 37) = 1.97, *p* = 0.169, or Group, *F*(2, 37) = 1.39, *p* = 0.262. There were no interaction effects of Block × Group, *F*(2, 37) = 1.14, *p* = 0.331, CS × Block, *F*(1, 37) = 1.21, *p* = 0.279, and CS × Block × Group, *F* < 1. In line with our expectations, however, there was a CS × Group interaction, *F*(2, 37) = 5.93, *p* = 0.006, $\eta_{p}^{2}=0.\text{243}$, which suggests group differences in differential responding. Follow-up *t*-tests, comparing the two CSs on averaged responding during the test phase, showed evidence for differential responding in group CS-only, *t*(15) = 3.87, *p* = 0.002 (α = 0.004), *d_z_* = 0.968, but not in the other groups, *t* ′s < 1.

### US Aversiveness

**Figure 4:**
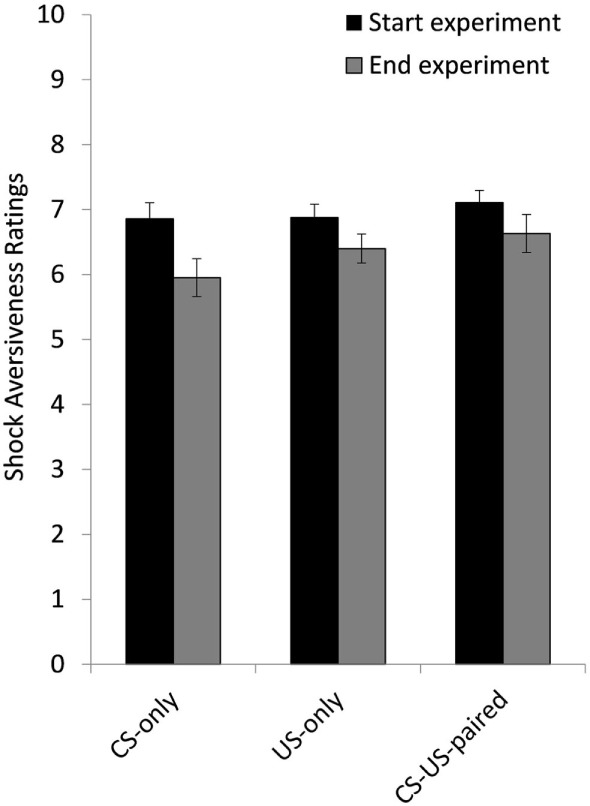
Mean ratings of shock aversiveness at the start and at the end of the experiment for group CS-only, US-only, and CS-US-paired. Error bars represent SEMs.

A 2 (Time) × 3 (Group) ANOVA on the aversiveness ratings showed a main effect of Time, *F*(1, 57) = 19.81, *p* < 0.001, *d* = 0.258 and no Time × Group interaction, *F*(2, 57) = 1.10, *p* = 0.341, meaning that self-reported US aversiveness decreased from the start to the end of the experiment in all three groups (see Figure 4).

## Discussion

This study compared the effects of CS exposures (standard extinction procedure), US exposures and continued CS–US pairings on the contextual renewal of CS-elicited fear. Unexpectedly, the standard context renewal effect following CS exposures was not replicated in our SCR data, which impedes the interpretation of other results on this measure. However, there was evidence of renewal after CS exposures for the CS+ compared to an unpaired control stimulus (CS−) in our self-report US expectancy and FPS data. US exposures, on the other hand, were associated with differential increases in US expectancy ratings but not in FPS responses. First, these results confirm that a US-only procedure outperforms a CS-only extinction procedure in reducing physiological fear in a contextual renewal test [cf. Ref. (6)]. Second, and in line with the rationale of this study, the intact differential US expectancy ratings may suggest that the US-only procedure acted via changing the estimated US intensity, rather than the CS–US association (cf. the Fear = Contingency × Intensity formula). This is further suggested by the strikingly similar results from the CS-US-paired group, where the continuous CS–US pairings could only have fortified the CS–US association, if anything. However, at odds with this explanation, we found no direct evidence for US devaluation taking place, i.e., we did not observe reductions in the subjective intensity of the US nor in electrodermal responding to repeated US presentations. Clearly, the present study was preliminary and drawing conclusions about the mechanism(s) of action would be inappropriate at this stage.

The majority of contextual renewal studies in humans have used skin conductance reactivity as the physiological proxy of conditioned fear (36). The current study replicated the standard fear renewal effect in startle reflex modulation [see also Ref. (41)], a physiological measure that has sometimes been deemed a closer index of fear [e.g., Ref. (19, 20)]. As in our previous studies with skin conductance reactivity (6, 42), there was no clear generalization of the conditioned discrimination to the extinction context (B), due to increased startle responding to the non-conditioned control stimulus (CS−). Nevertheless, the gradual decline of startle responding over the course of extinction is indicative of extinction learning, possibly to both stimuli and followed by differential renewal in the original conditioning context (A). Corroborating the study by Alvarez et al. (41), these startle results provide evidence that the contextual renewal effect in human fear conditioning does not merely reflect contingency learning (19).

The interventions examined in the present study abolished renewal of the startle response. This is remarkable because contextual renewal is notoriously difficult to weaken in comparison to other return of fear conditions [spontaneous recovery, reinstatement; see Ref. (43)]. Surprisingly, however, the current results contrast with some conditioning studies in rats that did show intact renewal of fear following a US exposure treatment [habituation (44, 45)] and following continued CS–US pairings [inhibition with reinforcement (46)]. Of course, many differences exist between the experimental setups that could explain the different findings, including species differences, experimental parameters and fear measures. The most obvious difference with regard to the difference in results is the timing of the different experimental phases. The series of human studies have conducted the experimental phases (conditioning, intervention, renewal test) consecutively in one session, while the rat studies conducted the different phases in individual sessions on separate days. While it is known that habituation of an unconditioned response (the response elicited by the US itself) generalizes across contexts (47–49), it does recover with time [(50, 51); but see Ref. (47, 48, 52)]. Hence, it remains to be seen whether the results from the human studies survive a temporal delay, e.g., a 24-h retention interval. Nevertheless, the fact that the CS exposures (extinction) procedure is sensitive to contextual renewal even in a one-session experiment strongly suggests that different mechanisms are at play in the US exposure treatments.

One issue related to the interpretation of the startle EMG data merits particular attention. When simply focusing on the CS+/CS− differentiation, Figure 3 shows that CS exposures ere associated with renewal of differential responding, and US exposures and continued CS–US pairings were not. However, one might argue that in group CS-only there was selective renewal for the CS+ and that responses to the CS− were not elevated compared to responses given during the ITIs, whereas in group US-only responses during both CS+ and CS− were elevated with respect to ITI. Accordingly, it may be inferred that US exposures may have caused generalization of conditioned responding to the CS−. Alternatively, the relatively high responses to the CS− may well be explained by (non-specific) stimulus sensitization. During the intervention phase, groups CS-only and CS-US-paired were continuously exposed to the CSs, whereas group US-only was not. Arguably, then, sudden test exposures to the CSs in group US-only may have caused strong orienting responses. Such effects can be expected for the CS + as well as for the CS−, yet no differential responding was observed in this group. Therefore, an interpretation of the data in terms of abolished renewal in group US-only seems more plausible.

It remains unclear, however, what mechanism explains the absence of renewal after US exposures. Unexpectedly, none of our direct indices of US intensity support the hypothesis that US devaluation would be at play. Yet, of note, continued CS–US pairings were associated with (minimal) extinction of differential startle EMG responding, indicative of inhibition with reinforcement. Such inhibition with reinforcement has been attributed to habituation to the US [(53, 54); but see Ref. (46)]. Possibly, then, our self-report and SCR indices of US intensity were not sufficiently sensitive. Accordingly, several candidate mechanisms may be considered. First, fear reduction may have actually been driven by US devaluation through habituation to the US, which, unlike extinction learning, generalizes to other contexts (47–49). Second, shock administrations in the acquisition context and the intervention context may have increased the similarity between both contexts, leading to easier generalization of US devaluation effects across contexts. If so, ABC or AAB setups may yield different results. Third, the gradual character of US devaluation via US exposure (the US is gradually perceived as less intense) may have reduced contextual influences. Extinction research suggests that a context-dependent extinction memory (CS-noUS) coexists with the more context-independent acquisition memory, resulting in contextual fear renewal (14). However, when the extinction rate is slowly increased (i.e., when reinforcement is gradually rather than suddenly decreased), a more robust fear decrease (no spontaneous recovery or reinstatement) occurs (55, 56). Possibly, gradual changes, like habituation effects, decrease context-dependency of the new created (extinction or US) memory or alternatively even change the original memory instead of constituting a new one. Of course, these explanations are purely speculative in light of the lack of evidence at present.

Exposure treatments are typically viewed as the clinical analog to the CS-only extinction procedure, but US exposures and even continued CS–US pairings may be involved as well. For example, a patient may have developed a driving phobia after experiencing a panic attack (US) while driving (CS). Exposure treatments will involve exposures to driving (CS), to the unpleasant sensations of panic attacks (US) and, ideally, to driving during a panic attack (CS–US) [e.g., Ref. (57, 58)]. Furthermore, the panic attack itself may function as a CS, as it is often associated with an even stronger US, e.g., fainting, “going crazy” or dying. This example shows that the CS/US dichotomy is not so clear in clinical practice and that exposure treatments will likely be a combination of CS-only, US-only, and CS–US exposures. It follows that preclinical fear reduction research may benefit from incorporating both US exposures and CS exposures in the experimental model for exposure-based treatments of anxiety.

There are a number of limitations to the current study. First, in the CS-only extinction group no renewal was observed in skin conductance ratings. The timing of probe administrations and the SCR window differed from other studies that combined measures of FPS and SCR (41), which may have caused interference of probes with the SCR (59). A recent study addressing the consequences of simultaneous employment of multiple fear measures demonstrated that the presentation of startle probes significantly interferes with the expression of fear through SCRs (59). Specifically, an experimental setup with startle probes (vs. without startle probes) was associated with relatively weak differential SCRs. The authors speculated that attenuated discrimination may have resulted from the aversive probe functioning as a secondary US, thereby interfering with safety learning to the CS−. There are only a few other studies on ABA renewal that combined FPS and SCR. Alvarez et al. (41) successfully demonstrated ABA renewal in both measures. Critically, perhaps, probes were presented during 75% of acquisition trials (vs. 100% in the current study) and during 83% of extinction trials (vs. 100% in the current study), which may have resulted in limited interference with differential acquisition of SCRs. Soeter and Kindt (60), also demonstrating ABA renewal in FPS and SCR, presented probes during all CS trials. However, these authors employed an instructed fear-learning paradigm in which CS–US contingencies were made explicit prior to conditioning, and such paradigm may be less susceptible to interference by inclusion of startle probes (59). Thus, different methodological approaches may explain the discrepancy between the present null findings and the results reported in the literature (6, 37, 60). Alternatively, failure in demonstrating ABA renewal of SCR is not uncommon [e.g., Ref. (61, 62)], and the current result may simply reflect a chance finding.

Second, we did not find direct evidence of US devaluation during the US exposure interventions. US aversiveness ratings (how unpleasant is the shock on a scale from 1 to 10) did not strongly decrease from the start to the end of the experiment. Possibly, a different measure [e.g., US costs (18)] may be a more comprehensive and sensitive measure of US intensity. Likewise, SCR did not decrease significantly over repeated US-only or paired CS–US trials during intervention. Strong reductions in unconditioned psychophysiological responses might require more repetitions of the US or CS–US [(45, 63); but see Ref. (6)]. Third, CSs were neutral shapes and electrical stimulation (US) was only mildly aversive. It is unclear to what extent these findings generalize to situations that involve fear-relevant CSs and very intense USs as is often the case in traumatic experiences. Fourth, because CS presentations were always combined with the shock prediction scale, the US-only group was not matched to the other groups with respect to the total number of scale exposures. Fifth, we found only weak evidence for fear decrease in FPS during the continued CS–US pairings. Typically, inhibition with reinforcement has been observed following massed CS–US pairings [dozens or hundreds (46, 54)]. This suggests that increasing the number of exposures may further maximize the context-independent fear reduction effects obtained with US- and CS–US exposures in the current study.

## Ethics Statement

This study was carried out in accordance with the recommendations of the Social and Societal Ethics Committee of KU Leuven with written informed consent from all subjects. All subjects gave written informed consent in accordance with the Declaration of Helsinki. The protocol was approved by the Social and Societal Ethics Committee of KU Leuven.

## Author Contributions

All authors listed have made substantial, direct, and intellectual contribution to the work and approved it for publication.

## Conflict of Interest Statement

The authors declare that the research was conducted in the absence of any commercial or financial relationships that could be construed as a potential conflict of interest.
